# COVID-19 Still Surprising Us—A Rare Movement Disorder Induced by Infection

**DOI:** 10.3390/brainsci12121733

**Published:** 2022-12-17

**Authors:** Carmen Adella Sirbu, Diana Popescu, Ion Stefan, Constantin Stefani, Marian Mitrica, Daniela Anghel

**Affiliations:** 1 Department of Neurology, ‘Dr. Carol Davila’ Central Military Emergency University Hospital, 010242 Bucharest, Romania; 2 Department of Medico-Surgical and Prophylactic Disciplines, Titu Maiorescu University, 031593 Bucharest, Romania; 3 Department of Infectious Diseases, ‘Dr. Carol Davila’ Central Military Emergency University Hospital, 010242 Bucharest, Romania; 4 Department No. 5, University of Medicine and Pharmacy “Carol Davila”, 050474 Bucharest, Romania; 5 Clinical Neurosciences Department, University of Medicine and Pharmacy “Carol Davila” Bucharest, 050474 Bucharest, Romania; 6 Department of Internal Medicine, Central Military Emergency University Hospital, 010242 Bucharest, Romania

**Keywords:** stiffness, reversible, rhabdomyolysis, stiff-person syndrome, COVID-19

## Abstract

Background: Many neurological manifestations are part of COVID-19 infections, including movement disorders, but a clinical picture closely resembling stiff-person syndrome has not yet been described. Case presentation: We report a case of a stiff-person-like syndrome in the context of COVID-19 infection. A 79-year-old woman, with no prior history of diseases, presented global reversible stiffness associated with SARS-CoV-2 infection. We aim to shed light on several particularities regarding this clinical picture and its evolution in close relationship with the infectious disease progression, with full regression of symptoms and signs once the infectious process ceased. The impairment of speech and motility caused the wrong diagnosis of stroke in the Emergency Room. In addition, we would also like to emphasize the concomitant rhabdomyolysis, closely linked to the grade of muscle rigidity. Conclusions: We would like to raise awareness regarding this clinical setting and its association with SARS-COV-2 infection, to aid in its future recognition and management. To our knowledge, this is the first case of a stiff-person-like syndrome to be described in association with COVID-19 infection.

## 1. Background

Since the identification of the novel coronavirus in 2019, named Coronavirus 2019 (COVID-19) by the World Health Organization, and the pandemic’s onset, the understanding of the disease has been evolving. A lot of progress has been made in characterizing the pathogen, disease pathogenesis, clinical picture, and courses of treatment, but its spectrum of clinical manifestations is still a subject of debate and a starting point for further research [1].

This is particularly true regarding the neurologic manifestations associated with this type of viral infection. Neurological events linked to COVID-19 infection range from mild to severe, life-threatening complications [2]. Myalgia, headache, dizziness, encephalopathy, anosmia, and dysgeusia appear to be more common, while stroke, seizures, sensory deficits, motor deficits, ataxia, and movement disorders have also been reported, but with a lower prevalence [3,4]. The musculoskeletal system is also known to be affected, with myositis and rhabdomyolysis also being described [5,6].

Stiff-person syndrome (SPS), a progredient disorder, with muscle spasms and painful episodes, has some triggers such as noise, touch, or even emotional distress [7]. In the classical variant of the syndrome, the affected muscles are the axial and limb muscles, the latter mostly in a proximal fashion. Patients present with truncal stiffness, abnormal postures, difficulty in walking, and a wide-base gait pattern [8]. The etiology of the syndrome still has not been identified, but it appears to be of autoimmune origin. There is a tendency toward co-association with other autoimmune diseases such as type 1 diabetes, pernicious anemia, vitiligo, and thyroiditis. In addition, some antibodies may be found in the patient’s serum (anti-glutamic acid decarboxylase-GAD) [9]. Other types of antibodies may also be found [10]. The syndrome has also been described in the context of paraneoplastic manifestations, usually associated with breast cancer or with lung small cell cancer [11]. A series of cryptogenic cases have also been described, in which the patients were seronegative, and as such, a link between immunological mechanism and the specific neurological manifestation could not be found [12]. As a general rule, stiff-person syndrome appears to be more frequent in women than in men [13].

We aim to present a case with a clinical picture closely resembling stiff-person syndrome occurring in the context of infectious disease, but with several particularities, the most important being the resolution of symptoms once the infection was abolished. In addition, we searched the literature for similar cases but we found similarities only for other infections which we briefly summarized.

## 2. Case Presentation

We present the case of a 79-year-old woman, with no prior history of neurological disease, no history of anti-SARS-COV-2 vaccination, and known to have chronic venous insufficiency. The patient presented in the emergency department with episodes of confusion, and speech and walking difficulties. Symptom onset was 2 days prior to admission. Due to her state, the setting of an acute stroke was taken into an account, a possibility that was later ruled out. For this reason, she was admitted to the neurology department and not in the infectious disease–COVID-19 designed area, although a rapid severe acute respiratory syndrome coronavirus 2 (SARS-COV-2) antigen test returned positive. The oxygen saturation level in the emergency department was between 90% and 92%. Her family reported fatigue and body temperature slightly above normal in the last 4 days, and nausea and vomiting. 

A neurological examination revealed a state of confusion, partially oriented to time, and her speech was difficult, slow, fragmented, slurred, and hard to understand. She had no signs of meningeal irritation and had general rigidity and hyperreflexia in both the upper and lower extremities. Muscle strength was preserved but she had difficulties in changing position and sitting up. The patient also experienced gait difficulties, as she could not walk unassisted. She experienced pain during passive movements of her limbs and also while attempting to walk, which she was reluctant to do. Spasms were predominantly affecting the axial and the proximal muscles of the lower limbs.

**Figure 1:**
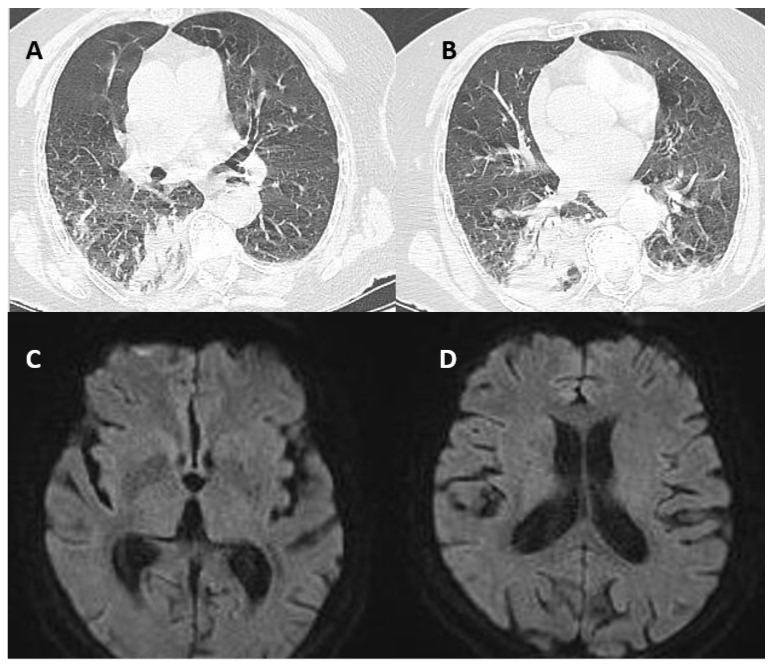
Imaging (**A**–**D**). Lung computed tomography shows bilateral areas of alveolar condensation and adjacent pleural effusion (**A**,**B**); Brain MRI-(DWI)—No areas of restricted diffusion were found (**C**,**D**).

Blood work showed leucopenia (3,81k/microliter), hyponatremia (134 mmol/L), and hyperfibrinogenemia (627 mg/dL). Erythrocyte sedimentation rate was 44 mm/1 h, Creatine Kinase (CK) was elevated (253 U/L), Creatine-kinase MB isoform was normal, and C-reactive protein and procalcitonin were also in the reference range. The lung Computer tomography (CT) indicated an area of alveolar condensation in the inferior right lobe, and also on the left, but of lower amplitude, with bilateral adjacent pleural effusion (Figure 1A,B). CT scan and Magnetic Resonance Imaging (MRI) of the brain were normal (Figure 1 C,D). Oral antiviral treatment was started with Molnupiravir 1600 mg daily alongside oxygen therapy. In evolution, a blood test showed leukocytosis (16 k/mL) and elevated inflammation markers in a crescent trend (C-reactive Protein was 181 mg/dL on day 4 and Procalcitonin 2.04 ng/mL). A set of blood cultures were taken but did not detect any microorganisms. Urine culture detected *E. Coli* (>100,000 cfu/mL). Intravenous antibiotic therapy with Linezolid (according to the antibiogram) 500 mg every 24 h was associated on day 3 with clinically and biologically favorable outcomes. Creatin-kinase enzyme correlated well with the grade of rigidity, as described in Table 1. There were no other biochemical and hematological relevant values. Antineoplastic markers were negative and other immunological tests including serum anti-Glutamic Acid Decarboxylase (GAD) antibodies as well. Although with certain difficulties regarding the epidemiological state (COVID-19 positive), an electromyographic test was performed on day 4. Electromyography showed characteristic continuous motor activity in agonists and antagonist muscles, with unmodified morphology. Nerve conduction studies were normal. The outcome was favorable: upon discharge (seven days later) there was no rigidity and no pain during passive mobilization. The patient regained full autonomy and could walk unassisted. She was fully oriented to time and space. Given the suggestive clinical picture and the electromyographic-specific findings, the clinical and paraclinical settings were highly suggestive of stiff-person syndrome. The absence of GAD antibodies and complete regression of her spasticity are unusual for SPS. The hypothesis of acute stroke was also taken into account upon admission due to speech and gait difficulties. The patient presented for a follow-up 6 months later and the neurological exam was normal. 

## 3. Discussion

Glutamic acid decarboxylase (GAD) antibodies described in stiff-person syndrome are targeted against GAD, one of the enzymes involved in the synthesis of the inhibitory neurotransmitter Gamma-Aminobutyric Acid (GABA). Reduction in GABA-ergic activity in the central nervous system translates into a reduction in the inhibitory signaling pathways, with the continuous firing of action potentials in the muscle fibers, so continuous uncoordinated contraction of agonist and antagonist muscles. Although anti-GAD antibodies are typically associated with stiff-person syndrome, a series of different antibodies have been described to occur in relationship with the disease. For example, in the paraneoplastic variant of the syndrome, anti-amphiphysin antibodies or antibodies against GABA receptor complex-associated proteins have proven to play an important role. The mechanism by which such antibodies appear is still unclear. The idea of molecular mimicry has been postulated, especially linked to malignancies or infectious diseases [14]. This is particularly important given the case we presented, of stiff-person-like syndrome associated with COVID-19 infection. 

To our knowledge, this is the first description of reversible global muscle rigidity, including limb and axial muscles, with superimposed muscle spasms in the context of COVID-19 infection. We searched the literature for similar cases that may have also been triggered by infectious diseases. We identified a series of cases of stiff-person syndrome or stiff-person-like syndromes (partial stiff-person syndrome) associated with West Nile Virus, HIV infection, borreliosis, hepatitis C virus (HCV) infection, each with specific characteristics as shown in Table 2 [15,16,17,18,19]. 

Further exploring the variant of cross-reactive immunity induced by an infectious agent, common epitopes have been found between West Nile Virus and GAD, which support the mechanism of the aberrant immune response against host proteins. In addition, it has been shown that in the setting of SPS or partial stiff-person syndrome (PSPS) that is triggered by an external pathogen, the course of the disease may be favorable, perhaps as the infectious process decreases or even ceases and the immune system of the host is no longer exposed to the cause, the autoimmunity inducing external antigen. The case cited in the literature of SPS as an occurrence following West Nile fever had no therapeutic benefit from specific treatment (pathogenic or symptomatic), instead responded very well to agents targeting the aberrant immunological pathways, and intravenous immunoglobulins administration. The clinical response was persistent over time, and the patient remained only mildly symptomatic [15]. In another case, we emphasized Lyme disease, which clinically presented as PSPS and showed a favorable clinical response to specific antimicrobial therapy [16,17]. Given the cases cited and taking into consideration the spectrum of immune-induced reactions already described in the context of COVID-19 infection, we hypothesize a possible cross-reactivity between COVID-19 antigen and some hosts antigens closely linked to the inhibitory neurotransmitter GABA, either directly, or by intermediary hosts proteins which interfere with GABA metabolism or receptor complex [20]. This hypothesis is in need of further exploration, but we would like to raise awareness of this specific clinical entity, as we believe it may be more common than it is currently perceived. It is rather unrecognized, undiagnosed, or mistaken for something else.

A few cases of stiff-person-like syndrome have been described as related to panhypopituitarism. The lack of response to the specific symptomatic treatment that is currently being used in the classical variant of SPS, and in contrast, the prompt clinical improvement after hormonal substitution strongly suggests the pathogenic link between the neuromuscular manifestation presenting with muscle stiffness and endocrine disturbances. The presumed pathogenesis is believed to be glucocorticoid deficiency with consecutive hyponatremia which, in turn, affects the muscular Na/K pump [21,22,23]. 

Furthermore, we would like to discuss another particularity of the case presented. In SPS, muscular enzymes are usually normal, or slightly above the maximum threshold. In the presented case, high values suggest an important simultaneous process of rhabdomyolysis [24,25]. Also, the CK values correlated well with the grade of muscle rigidity, as shown in Table 1. Creatine Kinase is usually elevated in myocardial infarction, acute kidney injury, renal colic, inflammatory myopathies, and immune-mediated necrotizing myopathies. The patient had no acute kidney injury, and the CK-Mb fraction was between normal limits [26]. Rhabdomyolysis is known to be linked to viral illnesses, including COVID-19 infection. Mechanisms are unclear; it is thought to appear either by the direct viral invasion of myocytes or by immunological mechanisms, cross reactivity, systemic inflammatory immune response, and cytokine storm [25]. The similar cases we found cited in the literature also reported high CK values [15,16].

We ruled out other disorders that mimic stiff-person syndrome as: other forms of myopathy, muscular dystrophies, tetanus, parkinsonism, dystonia, neuroleptic malignant syndrome, serotonin syndrome, Isaac syndrome, myelopathies, ankylosing spondylits, encephalitis, leokodistrophies [7].

A possible/probable functional motor disorder (FMD) has been described either during COVID-19 or after COVID-19 vaccination [27,28]. FMD is very common and it can manifest at any age with various phenotypes [29,30]. We found no inconsistency during the examination. We tried performing several distraction methods, but we did not identify any incongruence in the patient’s motor behavior and aspect of gait. In addition, the abnormal EMG result oriented against a possible functional disorder.

We believe this case is relevant as it emphasizes a particular clinical picture that has certain particularities regarding its management and treatment. As we have illustrated, such cases respond to specific causative treatment (specific antimicrobial therapy, hormonal substitution) or immunoglobulins and very little or not at all to specific GABA-targeting agents that are commonly used in the classical variant of the syndrome, such as benzodiazepines or muscle relaxants such as Baclofen, also known as a GABA modulating agents. Moreover, the non-recognition of this syndrome may cause a delay in the diagnostic process. This may lead to many complications, delaying advanced nursing measures.

## 4. Conclusions

To our knowledge, this is the first case of a stiff-person-like syndrome to be described in association with COVID-19 infection. Given the case we presented and the similar ones we found in the literature due to other infectious agents, our goal is to create a new, different perspective regarding the syndrome, beyond the classical, progressive variant associated with anti-GAD antibodies. It may be a more common syndrome than it is currently believed, which may be linked to more than one pathophysiological process. The stiff-person-like syndrome may be difficult to recognize and as a consequence, it may be misinterpreted as a completely different neurological entity, for example, acute stroke, as in the case we presented. The description of other neurological manifestations associated with COVID-19 infection not reported so far is of important value as it may aid in a prompt and correct diagnosis in future cases.

## Figures and Tables

**Table 1:** Creatine Kinase values during hospitalization compared to grade of rigidity.

Day of Hospitalization	CK Value	Grade of Rigidity
Day 1	253 U/L	+
Day 3	597 U/L	++
Day 4	12,030 U/L	++
Day 5	9797 U/L	+
Day 8	457 U/L	No rigidity

**Table 2:** Partial stiff-person syndrome linked to specific pathogens.

Associated Pathogen	Clinical Manifestation	Paraclinical Findings	Evolution
West Nile Virus	Increased muscle tone in left arm and legs (stiffness particularly in the arms and shoulder girdle area) Stiff appearance when walking Bradykinesia Hyperreflexia Plantar response extension bilaterally	Positive IgM and IgG antibodies to WNV in serum and CSF Positive serum anti-GAD antibodies Elevated Creatin-Kinase values CSF biochemistry, cellular count, and cultures Oligoclonal IgG antibodies in CSF Cervical and Brain MRI were normal Nerve conduction studies, repetitive nerve stimulation, and electromyography were normal	Complete resolution of a clinical picture after 3 months
Borrelia Burgdorferi	Pain and stiffness in the left leg; Spasmodic jerks and painful cramps in the left leg provoked by touch or loud noises; Difficulty walking with frequent falls; Reflex myoclonus in lower extremities which could be induced by touch, loud noises, touching or tapping the leg tendons or the bed	High CK values CSF analysis showed elevated proteins and elevated cell count Borrelia Burgdorferi-specific antibodies were found in serum and CSF Electromyography showed continuous motor activity in the muscles of the left leg Normal brain and spine MRI	Resolution of symptoms after 3 months
Hepatitis C Virus	Abnormal posture Motor and sensory deficits in upper and lower limbs Sphincter incontinence Diffuse painful muscle spasms in the extremities which could be induced emotional factors, noise and touch Hyporeflexia	HCV-RNA positive in serum Mild pleocytosis and elevated proteins at CSF analysis; Nerve conduction studies were normal Electromyography showed continuous muscle unit activity of agonist and antagonist muscles in the extremities; Normal brain and spine MRI	Unfavorable clinical outcome
*Brucella* spp.	Restricted vertical gaze movements; bilateral horizontal gaze-evoked nystagmus; diffuse spontaneous myoclonic spasms	Positive PCR test for Brucella in CSF Antiglycine receptor antibodies in serum and CSF; Normal brain and spine MRI; Continuous muscular activity on electromyography	Clinical improvement after 12 months

Caption: WNV = West Nile Virus; CSF = Cerebrospinal Fluid; MRI = Magnetic Resonance Imaging; PCR = Polymerase Chain Reaction.

## Data Availability

All relevant data have been presented in this manuscript and further inquiry can be directed to the corresponding authors.

## Abbreviations

COVID-19: coronavirus 2019; GAD: anti-glutamic acid decarboxylase; SARS-COV-2: severe acute respiratory syndrome coronavirus 2; CK: Creatine Kinase; CT: computer tomography; MRI: Magnetic Resonance Imaging; GABA: Gamma-Aminobutyric Acid; HCV: hepatitis C virus; WNV: West Nile Virus; CSF: Cerebrospinal Fluid.
